# Population-Based Resequencing of *APOA1* in 10,330 Individuals: Spectrum of Genetic Variation, Phenotype, and Comparison with Extreme Phenotype Approach

**DOI:** 10.1371/journal.pgen.1003063

**Published:** 2012-11-29

**Authors:** Christiane L. Haase, Ruth Frikke-Schmidt, Børge G. Nordestgaard, Anne Tybjærg-Hansen

**Affiliations:** 1 Department of Clinical Biochemistry, Rigshospitalet, Copenhagen, Denmark; 2 Copenhagen University Hospital and Faculty of Health and Medical Sciences, University of Copenhagen, Copenhagen, Denmark; 3 The Copenhagen General Population Study, Herlev Hospital, Herlev, Denmark; 4 The Copenhagen City Heart Study, Bispebjerg Hospital, Copenhagen, Denmark; 5 Department of Clinical Biochemistry, Herlev Hospital, Herlev, Denmark; University of Oxford, United Kingdom

## Abstract

Rare genetic variants, identified by in-detail resequencing of loci, may contribute to complex traits. We used the apolipoprotein A-I gene (*APOA1*), a major high-density lipoprotein (HDL) gene, and population-based resequencing to determine the spectrum of genetic variants, the phenotypic characteristics of these variants, and how these results compared with results based on resequencing only the extremes of the apolipoprotein A-I (apoA-I) distribution. First, we resequenced *APOA1* in 10,330 population-based participants in the Copenhagen City Heart Study. The spectrum and distribution of genetic variants was determined as a function of the number of individuals resequenced. Second, apoA-I and HDL cholesterol phenotypes were determined for nonsynonymous (NS) and synonymous (S) variants and were validated in the Copenhagen General Population Study (n = 45,239). Third, observed phenotypes were compared with those predicted using an extreme phenotype approach based on the apoA-I distribution. Our results are as follows: First, population-based resequencing of *APOA1* identified 40 variants of which only 7 (18%) had minor allele frequencies >1%, and most were exceedingly rare. Second, 0.27% of individuals in the general population were heterozygous for NS variants which were associated with substantial reductions in apoA-I (up to 39 mg/dL) and/or HDL cholesterol (up to 0.9 mmol/L) and, surprisingly, 0.41% were heterozygous for variants predisposing to amyloidosis. NS variants associated with a hazard ratio of 1.72 (1.09–2.70) for myocardial infarction (MI), largely driven by A164S, a variant not associated with apoA-I or HDL cholesterol levels. Third, using the extreme apoA-I phenotype approach, NS variants correctly predicted the apoA-I phenotype observed in the population-based resequencing. However, using the extreme approach, between 79% (screening 0–1^st^ percentile) and 21% (screening 0–20^th^ percentile) of all variants were not identified; among these were variants previously associated with amyloidosis. Population-based resequencing of *APOA1* identified a majority of rare NS variants associated with reduced apoA-1 and HDL cholesterol levels and/or predisposing to amyloidosis. In addition, NS variants associated with increased risk of MI.

## Author Summary

Rare genetic variants, identified by in-detail resequencing of loci, may contribute to complex traits. We used the apolipoprotein A-I gene (*APOA1*), a major high-density lipoprotein (HDL) gene, and population-based resequencing to determine the spectrum of genetic variants, the phenotypic characteristics of these variants, and how these results compared with results based on resequencing only the extremes of the apolipoprotein A-I (apoA-I) distribution. By resequencing *APOA1* in >10,000 Danes and genotyping an additional >45,000, we show that population-based resequencing of *APOA1* identifies a majority of rare genetic variants that together are relatively frequent: 0.27% of the population are heterozygous for nonsynonymous (NS) variants in *APOA1* that associate with substantial reductions in apoA-I and HDL cholesterol, and 0.41% are heterozygous for variants predisposing to amyloidosis. NS variants associated with a hazard ratio of 1.72 (1.09–2.70) for myocardial infarction (MI), largely driven by A164S, a variant not associated with apoA-I or HDL cholesterol levels. Resequencing only the extremes of the apoA-I distribution, between 79% and 21% of all variants are not identified; among these are variants previously associated with amyloidosis. These results provide direct evidence that rare NS variants in *APOA1* contribute to low apoA-I and HDL cholesterol levels, to susceptibility to amyloidosis, and to risk of MI in the general population.

## Introduction

Genome-wide association studies have identified multiple loci associated with complex traits and diseases, but until now common genetic variants (minor allele frequency >5%) at these loci only explain small proportions of the heritability [1], [2]. For example, the estimated heritability of high density lipoprotein (HDL) cholesterol in twin-studies is 50% [3], but the common alleles together or in combination explain less than 5–10% of the variation in plasma levels of HDL cholesterol [4]. Rare genetic variants (minor allele frequency <1%), which are identified by in-detail screening or resequencing of loci, may contribute to unravel this unexplained heritability [1], [2].

Apolipoprotein A-I (apoA-I) is the major protein component of HDL in plasma, and is a cofactor for lecithin∶cholesterol acyltransferase (LCAT), playing a key role in the so-called reverse cholesterol transport, i.e. the transport of cholesterol from peripheral tissues to the liver for excretion [3]. *APOA1* (MIM 107680) encodes a 267 amino acid prepropeptide, which is sequentially cleaved to yield the mature 243 amino acid protein. Mutations in apoA-I may associate with low levels of plasma HDL cholesterol and apoA-I due to defective LCAT activation or to amyloidosis, or to amyloidosis with only minor or no effects on apoA-I and HDL cholesterol levels [5]–[11]. However, at present we lack comprehensive information on the spectrum of genetic variants in this pleiotropic gene in the general population, on the phenotypic characteristics of such variants in individuals in the general population, and whether additional information is gained from resequencing a sample of the entire general population, rather than using an extreme phenotype approach, previously used by us and others [12]–[16].

In the first part of this study, the aim was to determine the spectrum and distribution of genetic variants in *APOA1* using a population-based resequencing approach. In the second part of the study, the aim was to determine the association of nonsynonymous (NS) and synonymous (S) variants in *APOA1* in the general population with plasma levels of apoA-I and HDL cholesterol, and with risk of myocardial infarction (MI). In the third part of the study, we compared results using an extreme apoA-I phenotype approach with results from the population-based resequencing. For these purposes, we resequenced *APOA1* in 10,330 participants in the Copenhagen City Heart Study (CCHS), and used the Copenhagen General Population Study (CGPS; n = 45,239 participants) to validate phenotypic results.

## Materials and Methods

### Subjects

Studies were approved by institutional review boards and Danish ethical committees (Nos. KF-100.2039/91, KF-01-144/01, H-KF-01-144/01) and conducted according to the Declaration of Helsinki. Informed consent was obtained from all participants. All participants were white and of Danish descent. No participants appeared in more than one of the two studies, permitting independent confirmation of the findings in each group.

#### The Copenhagen City Heart Study (CCHS)

The CCHS is a prospective study of the general population initiated in 1976–1978 with follow-up examinations in 1981–1983, 1991–1994, and 2001–2003 [17]–[19]. Individuals were selected based on the National Danish Civil Registration System to reflect the adult Danish population aged 20–80+ years. Data were obtained from a questionnaire, a physical examination, and from blood samples. Blood samples for DNA extraction were available on 10,330 participants attending the 1991–1994 and/or 2001–2003 examinations. Of these, 1,034 experienced an MI.

#### The Copenhagen General Population Study (CGPS)

The CGPS is a prospective study initiated in 2003 with ongoing enrollment [18], [19]. Participants were recruited and examined exactly as in the CCHS. At the time of genotyping, 45,239 had been included. Of these, 1,647 experienced an MI.

### Myocardial infarction

In both studies of the general population, diagnoses of MI (WHO International Classification of Diseases; ICD8:410, ICD10:I21-I22) were collected from 1977 through May 10^th^ 2011, and verified by reviewing all hospital admissions and diagnoses entered in the National Danish Patient Registry, and all causes of death entered in the National Danish Causes of Death Registry. A diagnosis of MI followed the changing definitions over time [20], [21].

### Study designs

**Figure 1:**
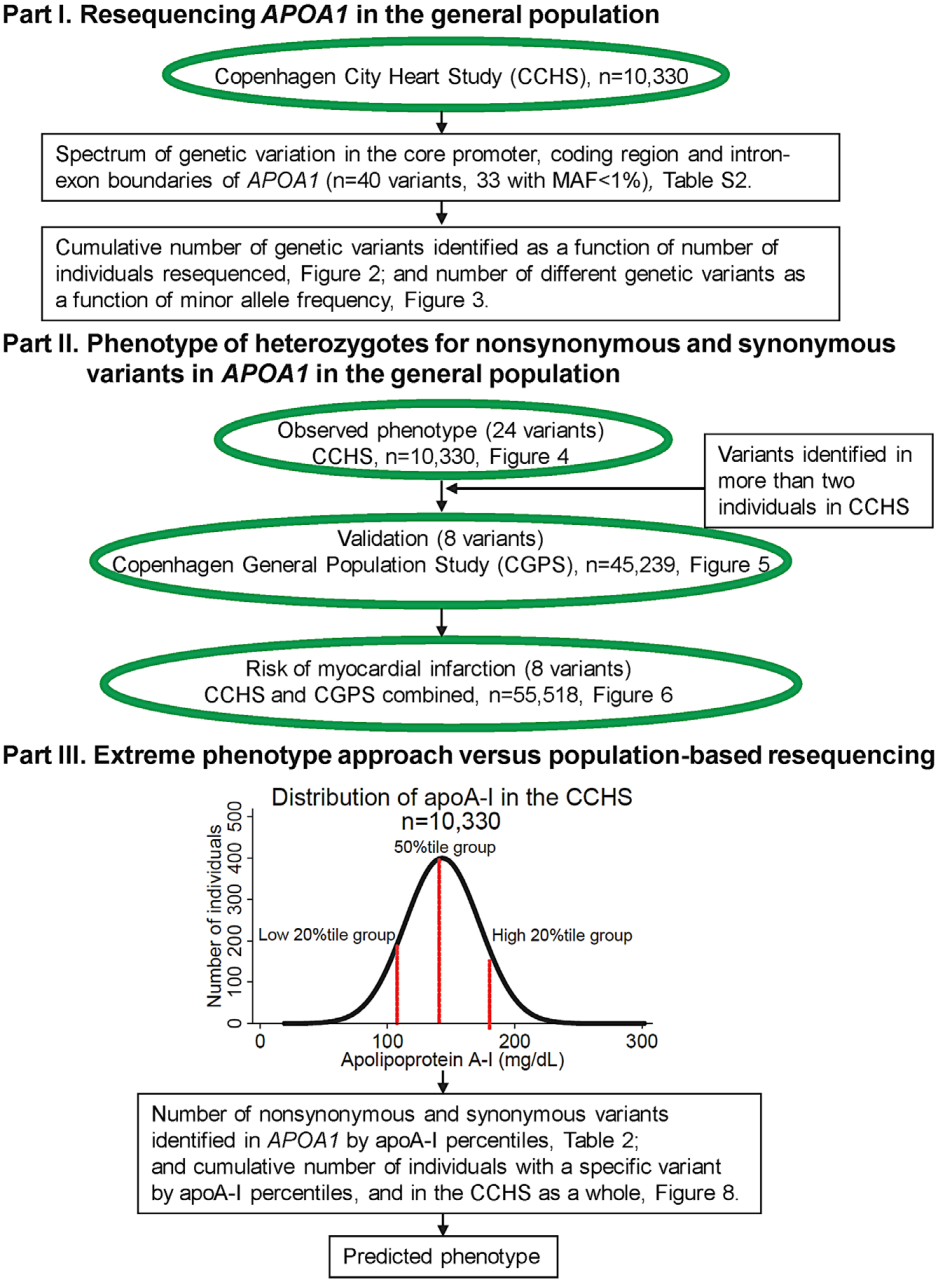
Study design. MAF = minor allele frequency.

As shown in flowchart (Figure 1).

#### Part I: Resequencing APOA1 in the general population

In the first part of this study, the aim was to determine the spectrum and distribution of genetic variation in *APOA1* in the general population. To this end, we resequenced the translated regions (exons 2–4) and exon-intron boundaries of *APOA1* in all 10,330 participants in the CCHS. We added data for the core promoter and the untranslated exon 1 (711 bp), for intervening sequences 2 (94 bp) and 3 (159 bp), and for the untranslated 3′end (175 bp) of the gene from a previous resequencing effort using the extreme phenotype approach, and including participants in the 1991–94 examination of the CCHS [12]. Thus, a total of 1,139 basepairs in non-coding regions were resequenced in 180 individuals only, likely resulting in an underestimation of very rare non-coding variants in these regions. We determined 1) the cumulative number of genetic variants identified as a function of the number of individuals resequenced, stratified by minor allele frequencies (MAFs) (MAFs>1%, 0.005%<MAF<1%, MAF = 0.005% = singletons), and 2) the number of different genetic variants identified as a function of the number of individuals with these variants, corresponding to the exact MAFs.

#### Part II: Phenotype of heterozygotes for nonsynonymous (NS) and synonymous (S) variants in APOA1 in the general population

In the second part of the study, the aim was to determine the association of NS and S variants in *APOA1* in the general population with plasma levels of apoA-I and HDL cholesterol, and with risk of MI. The association of NS and S variants with lipid and lipoprotein phenotypes was determined in the CCHS as a whole; for variants which occurred in more than two individuals in the CCHS, we validated the effect in the CGPS. The association between NS and S variants in *APOA1* and risk of MI was determined prospectively in the CCHS and CGPS combined. Finally, the results were compared with *in silico* prediction using PANTHER (www.pantherdb.org/), SIFT (http://sift.jcvi.org/), PolyPhen (http://genetics.bwh.harvard.edu/pph2/), and Pmut (http://mmb2.pcb.ub.es:8080/PMut/).

#### Part III: Extreme phenotype approach versus population-based resequencing

Using an extreme phenotype approach based on the apoA-I distribution in the CCHS, we compared the results using this approach with results from population-based resequencing. We first compared the number of NS and S variants identified in the extreme low and high percentiles (0–1%, 0–5%, 0–10%, 0–20%) of the distribution. We then predicted an assumed apoA-I phenotype based on the distribution of NS and S variants identified exclusively in the lowest and highest 0–1 percentiles up to 0–20 percentiles of apoA-I, an extreme phenotype approach previously used by us and others for a number of different genes [12]–[16]. We tested the validity of this prediction for all NS and S variants identified, by comparing with the apoA-I and HDL cholesterol phenotype determined in the CCHS as a whole.

### Gene screening and genotyping

We screened the translated region of *APOA1* in all 10,330 participants in the CCHS using four PCR fragments covering 119 bp upstream of exon 2, exons 2–4, and exon-intron boundaries (*APOA1* consensus sequence NC_000011.9) (Table S1). Mutational analysis was performed using a LightScanner (Idaho Technology Inc., Salt Lake City, UT, USA), followed by sequencing on an ABI 3730 DNA Analyzer (Applied Biosystems Inc., Foster City, CA, USA). NS and S variants identified in more than two individuals in the CCHS (K12K, S25S, S36A, F71Y, K107del, L144R, A164S, A190A), were genotyped in the CGPS using an ABI PRISM 7900HT Sequence Detection System (Applied Biosystems Inc., Foster City, CA, USA) and TaqMan-based assays.

### Lipids, lipoproteins, and apolipoproteins

Colorimetric and turbidimetric assays were used to measure nonfasting plasma levels of apoA-I, HDL cholesterol, total cholesterol, triglycerides, and apoB (Boehringer Mannheim, Mannheim, Germany and Konelab, Helsinki, Finland). LDL cholesterol was calculated using the Friedewald equation [22] when plasma triglycerides were ≤4.0 mmol/L, and otherwise measured directly (Thermo Fisher Scientific, Waltham, MA, USA).

### Other covariates

Body mass index was measured weight (kg) divided by measured height squared (m^2^). Lipid-lowering therapy was self-reported. Physical inactivity, drinking, smoking, hypertension and diabetes were dichotomized and defined as physical inactivity (less than 2–4 hours per week of light physical activity at leisure time), drinking (more than 1 drink per week), current smoking, hypertension (systolic blood pressure ≥140 mmHg or diastolic blood pressure ≥90 mmHg, and/or use of antihypertensive therapy), and diabetes (self-reported disease, current use of anti-diabetic medication, and/or nonfasting plasma glucose >11.0 mmol/L).

### Statistical analysis

We used Stata/S.E. 10.1. Two-sided p<0.05 was significant. χ^2^-tests evaluated Hardy-Weinberg equilibrium. To adjust for the effect of gender and age on absolute levels within studies, and for differences in absolute levels between studies, plasma levels of apoA-I and HDL cholesterol were converted to percentiles by gender and by age (in 10-year age groups) within each study, allowing for direct comparisons between percentiles for mutations both within and between studies (CCHS and CGPS). Mean apoA-I and HDL cholesterol percentiles in individuals with a specific mutation were compared with the mean percentile ( = 50^th^ percentile of the normalized distribution) within the CCHS or CGPS as a whole using a z-test [23]. Mann-Whitney U-test and Fisher's exact test compared, respectively, continuous and categorical variables between heterozygotes for different mutations and noncarriers. Number of NS and S variants identified among individuals with extremely low or high plasma levels of apoA-I were compared using Fisher's exact test. Risk of MI for heterozygotes for all NS (S36A, F71Y, K107del, L144R, A164S) and for all S (K12K, S25S, A190A) mutations genotyped in both studies, was determined prospectively in the CCHS and CGPS combined, using Cox proportional hazards regression models with age as time scale and delayed entry (left truncation) in 1977. Fify-one individuals with a previous MI were excluded from the risk analyses. Hazard ratios were adjusted for age and sex, or multifactorially for age, sex, diabetes, hypertension and smoking.

## Results

Study designs are shown as a flowchart in Figure 1.

### Part I: Resequencing APOA1 in the general population

**Figure 2:**
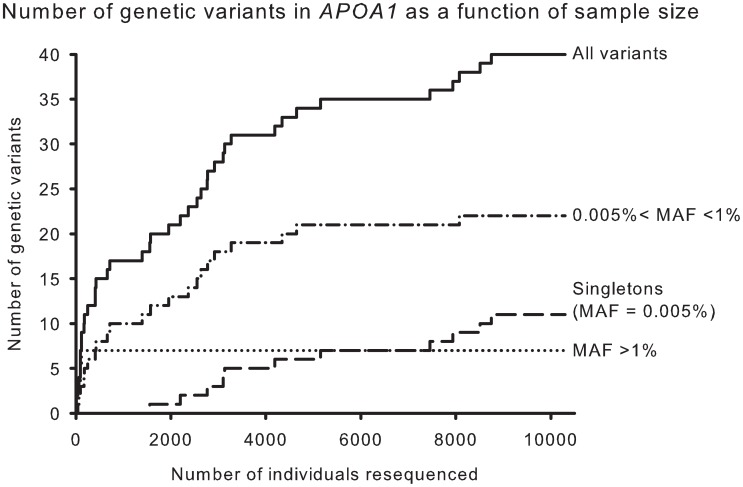
Cumulative number of genetic variants in *APOA1* as a function of number of individuals resequenced. Results are shown for all variants (solid) and for variants stratified by minor allele frequencies (MAFs), >1% (dotted), 0.005<MAF<1% (dash-dot), and MAF = 0.005% (long dash).

Resequencing *APOA1* in the CCHS identified a total of 40 genetic variants (Figure 2 and Table S2). Only seven variants (18%) had MAFs >1% (all in Hardy-Weinberg equilibrium, P-values: 0.12 to 0.82). Of these, none were in the coding region of *APOA1*, that is in exons 1–4 coding for the 267 amino acid prepropeptide. The cumulative number of genetic variants in *APOA1* as a function of the number of individuals resequenced showed that by resequencing fewer than 100 individuals all seven variants in *APOA1* with a MAF >1% were identified (Figure 2). In contrast, the number of genetic variants identified with a 0.005%<MAF<0.1% increased almost logarithmically reaching a plateau around 5,000 individuals, while the number of singletons (minor allele frequency = 0.005%) increased almost linearly.

**Figure 3:**
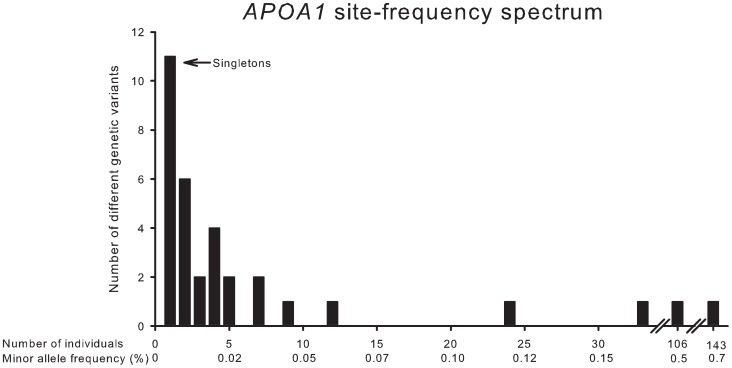
*APOA1* site-frequency spectrum. Number of different genetic variants identified in *APOA1* by resequencing as a function of individuals with each variant, and the corresponding minor allele frequencies.

In agreement with this, the number of different genetic variants identified in *APOA1* as a function of the number of individuals with each variant, corresponding to the MAF, showed that seventeen of 40 variants (43%) were exceedingly rare and identified in only one or two individuals in the CCHS (Figure 3).

### Part II: Phenotype of heterozygotes for nonsynonymous and synonymous variants in the general population

#### Observed phenotype

**Figure 4:**
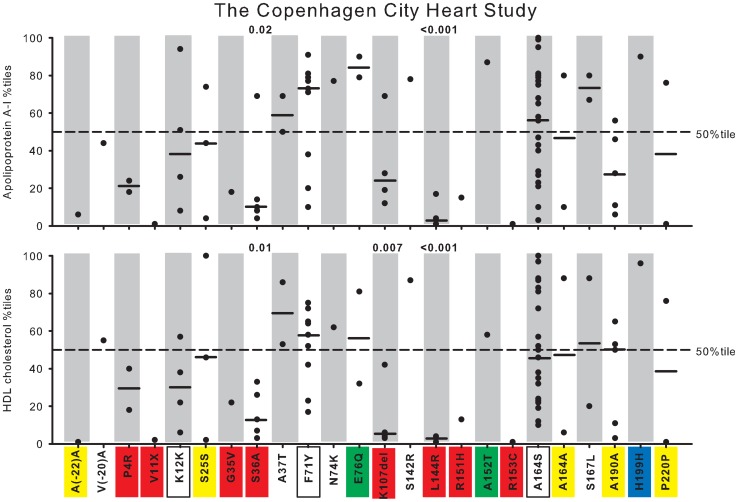
Plasma apoA-I and HDL cholesterol in percentiles for nonsynonymous and synonymous variants in *APOA1*. Data are from the Copenhagen City Heart Study (CCHS). Each dot represents an individual with a given genetic variant. Percentiles are corrected for gender and age. P-values according to z-test comparing mean apoA-I or HDL cholesterol percentiles in heterozygotes with individuals in the CCHS as a whole. Solid black lines = median percentiles. Dashed line = 50^th^ percentile. Red, nonsynonymous variants identified exclusively in the lowest 20 percentile of the apoA-I distribution in the CCHS using the extreme phenotype approach; yellow, synonymous variants identified exclusively in the lowest 20 percentile; green, nonsynonymous variants identified exclusively in the highest 20 percentile; blue, synonymous variants identified exclusively in the highest 20 percentile; white boxes, nonsynonymous and synonymous variants identified in both the lowest and highest 20 percentiles.

**Figure 5:**
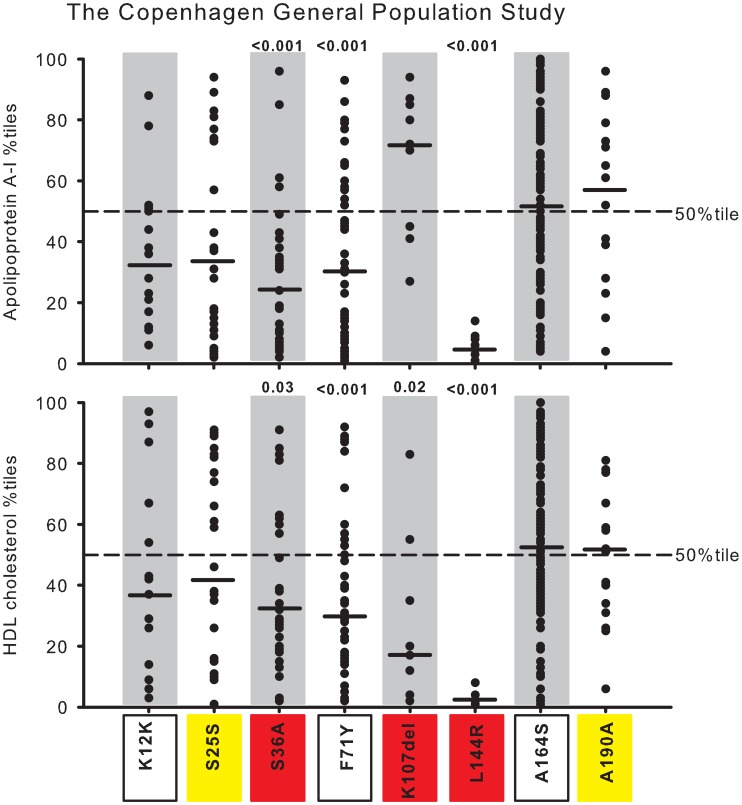
Plasma apoA-I and HDL cholesterol in percentiles for nonsynonymous and synonymous variants in *APOA1*. Data are from the Copenhagen General Population Study (CGPS). Each dot represents an individual with a given genetic variant. Percentiles are corrected for gender and age. P-values according to z-test comparing mean apoA-I or HDL cholesterol percentiles in heterozygotes with individuals in the CGPS as a whole. Solid black lines = median percentiles. Dashed line = 50^th^ percentile. Red, nonsynonymous variants identified exclusively in the lowest 20 percentile of the apoA-I distribution in the CCHS using the extreme phenotype approach; yellow, synonymous variants identified exclusively in the lowest 20 percentile; white boxes, nonsynonymous and synonymous variants identified in both the lowest and highest 20 percentiles.

In Figure 4, Figure 5, and Figure S1, plasma levels of apoA-I and HDL cholesterol, for the individual mutations, are shown as percentiles for heterozygotes versus percentiles in the population as a whole. This corrects for the effect of gender and age on absolute plasma levels within and between studies, and for differences in absolute plasma levels of apoA-I and HDL cholesterol between studies, thus allowing for direct comparisons.

Seventeen NS (9 new) and seven S (6 new) variants in *APOA1* were identified in a total of 80 individuals (0.77%) by the population-based resequencing of the CCHS (Table S2). Two NS variants, S36A and L144R, associated with reduced apoA-I and HDL cholesterol with median levels of apoA-I at, respectively, the 10^th^ and 3^rd^ percentiles, and with corresponding HDL cholesterol levels at the 14^th^ and 2^nd^ percentiles, compared with the apoA-I and HDL cholesterol distributions in the CCHS as a whole (P-values: 0.02 to <0.001) (Figure 4, in red). K107del associated with a median HDL cholesterol level at the 5^th^ percentile (P = 0.007) (Figure 4, in red), without a corresponding reduction in apoA-I, while P4R appeared to associate with both low apoA-I and HDL cholesterol levels (Figure 4, in red). None of the other variants associated with apoA-I or HDL cholesterol levels, although the singletons V11X, G35V, R151H and R153C all were within the lowest apoA-I and HDL cholesterol percentiles (Figure 4, in red), and the singleton A152T was in the highest apoA-I percentiles (Figure 4, in green).

#### Validation of observed phenotype

To further validate the associations between *APOA1* genotype and plasma levels of apoA-I and HDL cholesterol observed in the CCHS, we genotyped all eight NS and S variants identified in more than two individuals in the CCHS (MAF >0.01%; K12K, S25S, S36A, F71Y, K107del, L144R, A164S, A190A) in the 45,239 participants in the CGPS. In this study, S36A associated with median apoA-I and HDL cholesterol levels at, respectively, the 24^th^ and 29^th^ percentile, and L144R associated with corresponding median levels at the 5^th^ and 2^nd^ percentiles (P-values 0.03 to <0.001; Figure 5, in red), confirming the results from the CCHS (Table S3, CCHS versus CGPS comparing relative percentile reductions: P-values from 0.90 to 0.07). We also confirmed that the deletion of one amino acid at position 107 (K107del) associated with a median HDL cholesterol level at the 17^th^ percentile (P<0.05), without an effect on apoA-I levels, although apoA-I levels were higher in the CGPS (Figure 5, in red) (Table S3, CCHS versus CGPS: P-values 0.31 for HDL cholesterol and 0.03 for apoA-I). In addition, F71Y associated with lower than average apoA-I (median 31^st^ percentile) and HDL cholesterol levels (median 29^th^ percentile) in the (well-powered) CGPS (P-values <0.001), but not in the (less-powered) CCHS (compare Figure 4 and Figure 5, white and boxed; Table S3, CCHS versus CGPS: P-values 0.02 and 0.04). The NS variant A164S and the S variants K12K (both white and boxed), S25S (yellow) and A190A (yellow) did not associate with apoA-I or HDL cholesterol levels, also in agreement with results from the CCHS (Figure 4 and Figure 5 and Table S3, CCHS versus CGPS: P-values from 0.09 to 0.94). A comparison of the median differences between the CCHS and the CGPS in relative percentile values (50^th^ percentile in population minus percentile for heterozygotes) and in absolute plasma levels of apoA-I and HDL cholesterol (noncarriers minus heterozygotes) for the individual mutations are shown in Table S3.

Potential gender differences for apoA-I and HDL cholesterol percentiles among heterozygotes for the individual mutations in CCHS and CGPS combined are shown in Figure S1. Although the effect of some variants appeared to differ between genders, there were no consistent patterns of gender heterogeneity.

Characteristics of heterozygotes for each of the four mutations associated with reductions in plasma levels of apoA-I and/or HDL cholesterol levels are shown for the CCHS and CGPS in Table 1. Comparing heterozygotes with noncarriers, only plasma levels of apoA-I and HDL cholesterol (mirrored in total cholesterol for L144R) were consistently different in both studies, suggesting that the effect of genotypes on these two parameters were unconfounded by other measured characteristics. In the CCHS and CGPS, the absolute reductions in plasma levels of apoA-I ranged from 14 mg/dL (F71Y) to 39 mg/dL (L144R), and in HDL cholesterol from 0.3 mmol/L to 0.9 mmo/L for the same variants (Table 1 and Table S3). For comparison, we have previously shown that the combination of two common variants in *APOA1*, −560A>C (MAF = 3.5%, tagging the haplotype-560A>C, −151C>T, *181A>G) and −310G>A (MAF = 16%), associated with minor increases in plasma levels of apoA-I and HDL cholesterol of up to, respectively, 9 mg/dL and 0.1 mmol/L in the 1% of individuals heterozygous for both variants [12].

**Table 1:** Characteristics of *APOA1* S36A, F71Y, K107del, and L144R heterozygotes and noncarriers in the Copenhagen City Heart Study (n = 10,330) and the Copenhagen General Population study (n = 45,239).

	Copenhagen City Heart Study					Copenhagen General Population study				
	Noncarriers	Heterozygotes				Noncarriers	Heterozygotes			
		S36A	F71Y	K107del	L144R		S36A	F71Y	K107del	L144R
	n = 10,308	n = 5	n = 9	n = 4	n = 4	n = 45,141	n = 29	n = 48	n = 9	n = 12
Age (years)	59 (45–69)	67 (38–37)	62 (59–68)	66 (52–70)	47 (44–62)	57 (47–67)	65 (56–71)^b^	52 (45–66)	50 (41–57)	57 (41–61)
Sex (F/M)	5,738/4,570	2/3	8/1	2/2	3/1	24,847/20,294	23/6^b^	32/16	4/5	8/4
Apolipoprotein A-I (mg/dL)	140 (123–161)	106 (106–120)^b^	162 (144–169)	120 (108–148)	101 (84–118)^b^	156 (139–176)	139 (131–162)^a^	142 (130–162)^b^	169 (151–172)	117 (91–125)^b^
HDL cholesterol (mmol/L)	1.5 (1.2–1.8)	1.1 (1.0–1.2)^a^	1.7 (1.5–1.8)	1.0 (0.9–1.4)^a^	0.8 (0.7–0.9)^b^	1.6 (1.3–2.0)	1.5 (1.3–1.8)	1.3 (1.1–1.7)^b^	1.1 (0.9–1.4)^a^	0.7 (0.6–1.0)^b^
Total cholesterol (mmol/L)	6.0 (5.1–6.9)	5.0 (4.2–5.7)	6.7 (6.2–6.9)	6.9 (6.4–7.9)	4.7 (4.2–5.4)^a^	5.6 (4.9–6.3)	5.8 (5.4–6.0)	5.3 (4.4–6.3)	5.7 (5.5–5.9)	5.3 (4.1–5.5)^a^
LDL cholesterol (mmol/L)	3.6 (2.9–4.4)	3.0 (2.2–3.8)	3.7 (3.6–4.5)	4.5 (4.2–5.3)^a^	3.2 (2.7–3.4)	3.2 (2.6–3.8)	3.3 (2.7–3.8)	3.0 (2.4–3.9)	3.3 (3.1–3.8)	3.4 (3.0–3.7)
Triglycerides (mmol/L)	1.5 (1.1–2.2)	1.8 (1.8–1.9)	1.7 (1.4–1.8)	2.2 (1.5–3.3)	2.2 (1.2–3.0)	1.4 (1.0–2.1)	1.4 (1.2–1.8)	1.6 (0.8–2.4)	1.3 (0.8–2.8)	2.0 (1.1–2.5)
Apolipoprotein B (mg/dL)	86 (71–103)	70 (64–90)	93 (89–107)	102 (96–119)	78 (69–107)	107 (88–131)	112 (96–128)	99 (83–145)	120 (102–144)	118 (99–142)
Body mass index (kg/m^2^)	25(22–28)	27(26–28)	26(22–28)	28(25–32)	27(24–29)	26 (23–29)	25 (22–28)	25 (23–27)	24 (22–26)	26 (24–28)
Lipid lowering therapy (%)	1	0	0	0	0	10	10	13	11	17
Physical inactivity (%)	64	80	56	25	75	52	48	58	67	42
Drinking (%)	57	80	56	25	100	32	28	26	56	25
Smoking (%)	47	20	67	50	25	22	17	28	22	2
Hypertension (%)	52	80	67	25	50	61	78	56	25	58
Diabetes (%)	4	20	0	0	0	4	0	4	0	0

Values are median (interquartile range) or percentage. Mann-Whitney U-test or Fishers exact test was used for continuous and categorical traits, respectively. Lipid-lowering therapy was self-reported. Physical inactivity, drinking, smoking, hypertension and diabetes were dichotomized and defined as physical inactivity (less than 2–4 hours per week of light physical activity at leisure time), drinking (more than 1 drink per week), current smoking, hypertension (systolic blood pressure ≥140 mmHg or diastolic blood pressure ≥90 mmHg, and/or use of antihypertensive therapy), and diabetes (self-reported disease, current use of anti-diabetic medication, and/or nonfasting plasma glucose >11.0 mmol/L). LDL cholesterol levels were calculated using the Friedewald equation if triglycerides were ≤4 mmol/L, but measured directly at higher triglyceride levels.

a P<0.05;

b P<0.01.

Finally, collapsing all NS and S variants genotyped in the CCHS (n = 24) (Table S4, top), or in both the CCHS and CGPS (n = 8) (Table S4, bottom), showed that both NS and S variants were associated with significant reductions in plasma levels of apoA-I and/or HDL cholesterol in the CGPS, and that these effects were more pronounced when A164S, a relatively frequent variant without effect on apoA-I or HDL cholesterol [13], was excluded from the analysis. Combining the two studies confirmed that S variants associated with reductions in apoA-I (P = 0.03), and showed that variants suspected of (A164S) or previously associated with amyloidosis (S36A, F71Y, K107del) tended to have higher levels of apoA-I and HDL cholesterol than variants associated with reduced LCAT activation (L144R) (Figure S2; P-values <0.01).

In summary, results for seven of the eight most common NS and S variants in the CCHS were confirmed by genotyping in the CGPS. In this very large study, an additional variant, F71Y, associated with low apoA-I and HDL cholesterol levels. Thus, taken together approximately 0.27% of individuals in the general population carry NS mutations in *APOAI*, which associate with substantial reductions in apoA-I and/or HDL cholesterol levels compared to noncarriers. Combining NS and S variants in both the CCHS and CGPS, suggested that S variants also were associated with reductions in apoA-I levels.

#### Contribution of rare and common variants to variation in plasma levels of apoA-I and HDL cholesterol

The correlation between plasma levels of HDL cholesterol and apoA-I was R = 0.83 in the CCHS, that is, 69% (R^2^ = 0.69) of the total variability in HDL cholesterol levels were explained by the linear relationship with apoA-I levels. Results were similar in the CGPS.

In the CCHS, common variants in the promoter and coding regions of *APOA1* (−560A>C MAF = 3.5%, tagging the haplotype-560A>C, −151C>T, *181A>G, and −310G>A MAF = 16%) contributed 0.4% (R^2^ = 0.004) to the total variability in plasma levels of apoA-I, and 0.2% to the total variability in HDL cholesterol; the corresponding contributions from rare variants were 10-fold lower, respectively, 0.03% and 0.04%. Contributions were similar in the CGPS.

#### Risk of myocardial infarction

**Figure 6:**
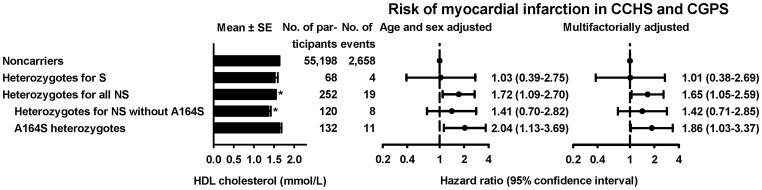
HDL cholesterol levels and risk of myocardial infarction for heterozygotes for synonymous (S) or nonsynonymous (NS) variants in *APOA1*. Hazard ratios by Cox proportional hazard regression models with age as time scale and delayed entry (left truncation) in 1977 based on the combined study of Copenhagen City Heart Study and the Copenhagen General Population. *P<0.01 comparing heterozygotes with noncarriers by Mann-Whitney U-test.

Risk of MI was determined for heterozygotes for S (n = 68) and NS (n = 252) variants in *APOA1* in the CCHS and CGPS combined (Figure 6). Hazard ratios for MI were 1.03 (95% CI:0.39–2.75) for heterozygotes for S variants, 1.72 (1.09–2.70) for heterozygotes for all NS variants, 1.41 (0.70–2.82) for NS variants without A164S, and 2.04 (1.13–3.69) for A164S alone, validating previous results for this variant based on the CCHS alone [13]. Results were similar after multifactorial adjustment for age, sex, diabetes, hypertension, and smoking.

#### Distribution, evolutionary conservation, and *in silico* prediction of functional effects

**Figure 7:**
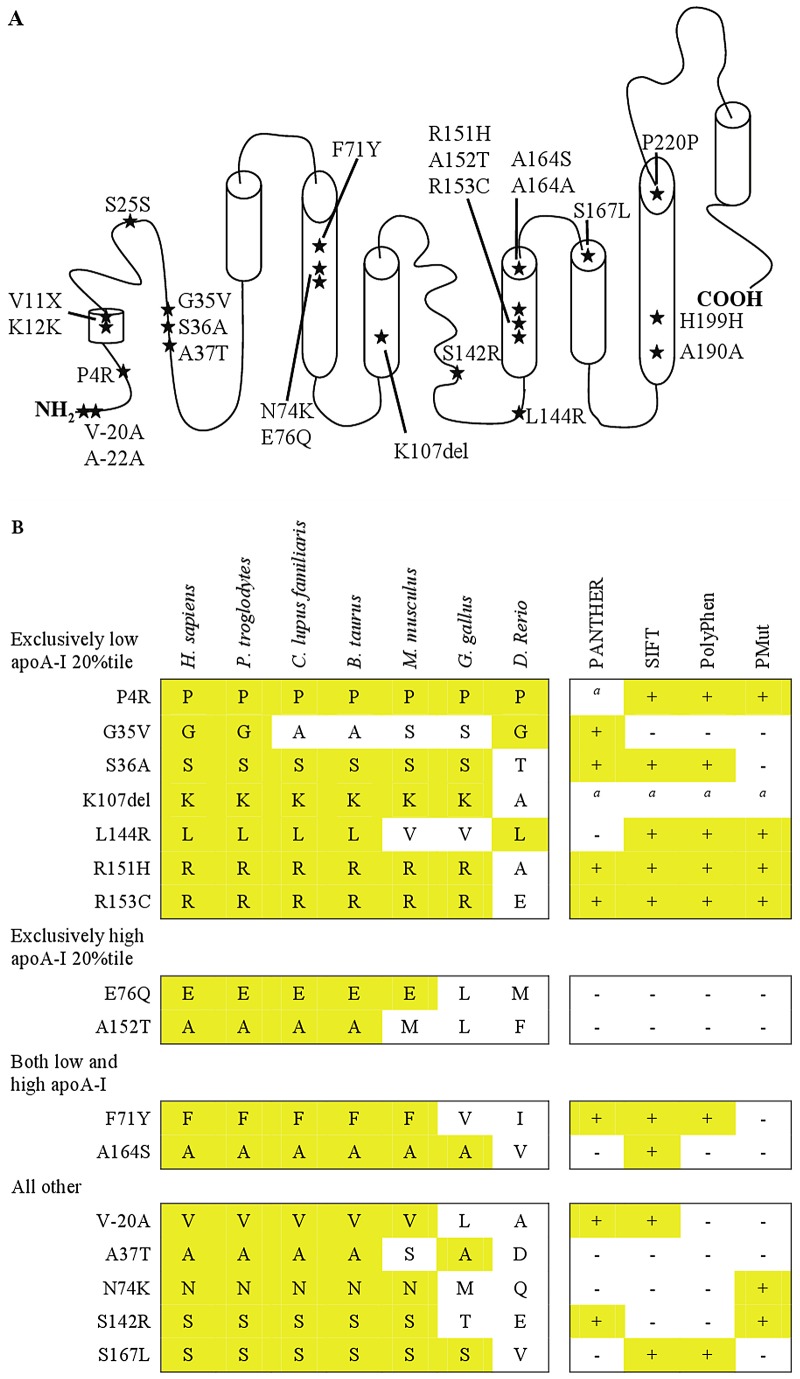
Nonsynonymous and synonymous variants in *APOA1*. A. The positions of all nonsynonymous and synonymous variants identified by resequencing *APOA1* in the Copenhagen City Heart Study (CCHS, n = 10,330) have been superimposed on the secondary structure of apoA-I. B. Evolutionary sequence conservation and predicted functional effects of nonsynonymous genetic variants in *APOA1*. Variants are divided into four groups, depending on whether they were identified exclusively in the lowest 20 percentile, the highest 20 percentile, or in both the lowest and highest 20 percentiles of the apoA-I distribution, using the extreme phenotype approach. The fourth group includes variants exclusively identified using the population-based resequencing approach. The truncation, *APOA1* V11X, is not included in the figure. The alignment includes human (*H. sapiens*), chimpanzee (*P. troglodytes*), dog (*C. lupus familiaris*), cow (*B. taurus*), mouse (*M. musculus*), chicken (*G. gallus*), and zebrafish (*D. Rerio*). Alignment by HomoloGene (www.ncbi.nlm.nih.gov/homologene/). *^a^*not possible to model. PANTHER: + = P-deleterious>0.5; − = P-deleterious<0.5 (www.pantherdb.org/). SIFT: + = affect protein function; − = tolerated (http://sift.jcvi.org/). PolyPhen: + = probably or possibly damaging; − = benign (http://genetics.bwh.harvard.edu/pph2/). PMut: + = pathological; − = neutral (http://mmb2.pcb.ub.es:8080/PMut/). Figure 7A was adapted from [6].

NS and S variants appeared evenly distributed throughout the protein, although no NS variants were identified more C-terminal than S167L (Figure 7A). Almost all NS variants were highly conserved (except G35V), and all variants identified exclusively in the lower 20^th^ percentile of apoA-I, including variants associated with low apoA-I and/or HDL cholesterol (P4R, S36A, L144R, F71Y – no prediction for K107del) in the CCHS and/or the CGPS, were predicted to affect function by at least three of four *in silico* programs (Figure 7B). In addition two singletons, R151H and R153C, in, respectively, the lowest 20^th^ and 1^st^ percentiles of the apoA-I distribution in the CCHS, were predicted to affect function by all four programs.

### Part III: Extreme phenotype approach versus population-based resequencing

#### Number of nonsynonymous and synonymous variants in *APOA1* by apoA-I percentiles

Comparing the number of NS and S variants identified exclusively in the extreme percentiles of the apoA-I distribution (0–1, 0–5, 0–10, 0–20, 0–50 percentiles) in the CCHS, revealed a predominance of variants in the low percentile versus the high percentile groups (Table 2; P-values: 0.004 to 0.02; comparing lowest versus highest 0–5 to 0–20 percentiles), suggesting that most variants were loss of function variants associated with low levels of apoA-I. Nineteen of a total of 24 variants (79%) (Table 2), were identified by resequencing individuals in the lowest and highest 20 percentiles of apoA-I, whereas resequencing the 0–1, 0–5 and 0–10 percentiles identified, respectively, five (21%), eight (33%), and twelve variants (50%).

**Table 2:** Number of nonsynonymous and synonymous variants identified in *APOA1* by apoA-I percentiles.

Extreme phenotype screened, %tile	Number of genetic variants							
	Total	Exclusively low apoA-I		Exclusively high apoA-I		Both low and high apoA-I		
		NS	S	NS	S	NS	S	P-value^a^
0–1	5	3	1	1	0	0	0	0.39
0–5	8	4	3	0	0	1	0	0.02
0–10	12	4	5	0	0	2	1	0.004
0–20	19	8	5	2	1	2	1	0.02
b0–50	24	7	1	5	1	5	5	0.79

a P-values by Fisher's exact test comparing nonsynonymous (NS) and synonymous (S) variants combined and identified exclusively in the low apoA-I or in the high apoA-I group. Values represent the cumulative number of genetic variants identified in the lowest and highest 0–1 percentile, 0–5 percentile and 0–10 percentile, 0–20 percentile and 0–50 percentile of the apoA-I distribution in the Copenhagen City Heart Study.

b The upper and lower 0–50 percentiles combined correspond to the total number of nonsynonymous and synonymous variants identified in the Copenhagen City Heart Study.

#### Predicted phenotype using extreme phenotype approach

**Figure 8:**
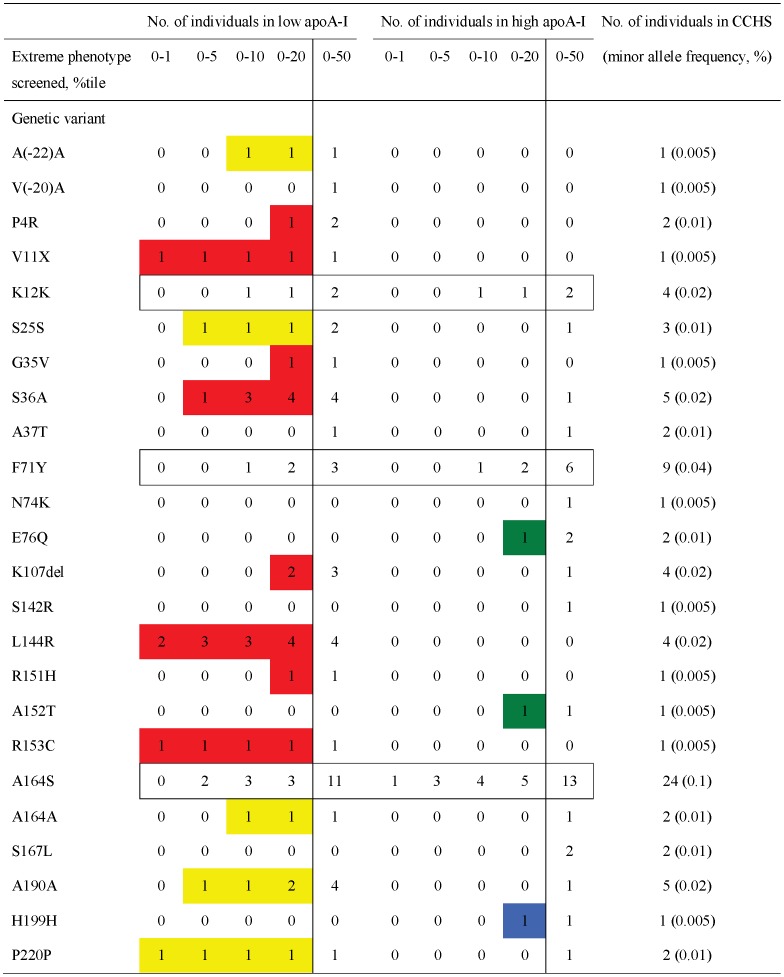
Cumulative number of individuals with a specific nonsynonymous or synonymous variant in *APOA1* by apoA-I percentiles in the Copenhagen City Heart Study. Red, nonsynonymous variants identified exclusively in the lowest 20 percentile of the apoA-I distribution using the extreme phenotyping approach; yellow, synonymous variants identified exclusively in the lowest 20 percentile; green, nonsynonymous variants found exclusively in the highest 20 percentile, blue, synonymous variants identified exclusively in the highest 20 percentile; white boxes, nonsynonymous and synonymous variants identified in both the lowest and highest 20 percentiles. In plain white, variants only identified by population-based resequencing, corresponding to the 20–80 percentile.

To determine whether the apoA-I and HDL cholesterol phenotype predicted from the distribution of the variants in the extreme 20 percentile groups (exclusively low, exclusively high or both) corresponded to the phenotype observed in the CCHS as a whole, we compared the results from the extreme phenotype approach with results observed from the population-based resequencing of the CCHS (compare Figure 4 with Figure 8 – same color code).

Based on the extreme phenotype approach and a 20 percentile cut-off, thirteen variants (Figure 8, NS in red and S in yellow) identified in 32 (0.31%) individuals in the CCHS were predicted to associate with low apoA-I and HDL cholesterol levels, three variants (Figure 8, NS in green and S in blue) identified in four (0.04%) individuals were predicted to associate with high apoA-I and HDL cholesterol, whereas the remaining variants identified in both extreme percentile groups (Figure 8, white boxed, K12K, F71Y, A164S) in 37 (0.36%) individuals were not predicted to affect apoA-I or HDL cholesterol levels. Five NS variants (V(-20)A, A37T, N74K, S142R, S167L, Figure 8 in plain white) in seven individuals (0.07%) were only identified by the population-based resequencing approach.

Comparing these results with results from the population-based resequencing showed that NS variants associated with low apoA-I and HDL cholesterol levels or with no effect in the CCHS were correctly predicted using the extreme 20 percentile approach (compare Figure 4 with Figure 8, variants in red and boxed white). However, the number of these variants identified depended on the extreme percentiles resequenced (Figure 8, compare 0–1 versus 0–20 percentiles). Synonymous variants predicted to associate with low apoA-I and HDL cholesterol were not correctly predicted (compare Figure 8 with Figure 4, variants in yellow). NS and S variants predicted to associate with high apoA-I and HDL cholesterol levels were mostly rare, and could therefore not be validated (Figure 4 and Figure 8, variants in green and blue). Using the extreme phenotype approach, between 19 (79%; 0–1 percentile) and 5 (21%; 0–20 percentile) of all variants were not identified at all depending on the extreme percentile resequenced, among these variants known to, or suspected of associating with amyloidosis (F71Y, A164S) [13], [24], and with risk of MI and early death (A164S) [13].

## Discussion

Using population-based resequencing of *APOA1* in 10,330 individuals allowed description of the spectrum and distribution of genetic variants in this gene. Our results showed that the vast majority of variants, including variants associated with apoA-I and HDL cholesterol phenotype, were individually rare, though collectively relatively common. These results are in complete agreement with results from two previous population-based resequencing studies of three other genes affecting, respectively, triglycerides and diabetes related traits [25], [26].

Novel findings, compared to previous population-based screenings of apoA-I using isoelectric focusing [27], [28], include: First, the number of NS variants identified and the number of heterozygotes for these variants were, respectively, 5–6 fold and 10–20 fold increased. Second, we showed that 0.27% of the population were heterozygous for variants associated with substantial reductions in apoA-I and HDL cholesterol levels, and 0.41% were heterozygous for variants previously associated with amyloidosis, although none had been diagnosed with this disease. In addition, S variants, not identified by isoelectric focusing, also associated with reductions in apoA-I levels in the CCHS and CGPS combined (n>55,000). Third, heterozygosity for NS variants in *APOA1* associated with a 2-fold increased risk of MI, largely driven by A164S, a variant not associated with apoA-I and HDL cholesterol levels. Finally, while these rare variants might have some effects on the extremes of the population distribution of apoA-I and HDL cholesterol and on levels in the individual, the contribution of both rare and common variants in *APOA1* to the total variation in plasma levels of apoA-1 and HDL cholesterol were very modest, respectively, 0.03% and 0.3%, in agreement with the very large number of genes found to associate with apoA-I and/or HDL cholesterol levels in genomewide association studies [29]. The effect of common variants on plasma levels were 10-fold higher than for rare variants, suggesting that rare variants in this gene do not contribute in any major way to the missing heritability on a population level.

An advantage of population-based resequencing is that the genetic variants identified can be tested against multiple phenotypes. This becomes especially important, if the gene under study has pleiotropic effects, i.e. affects multiple phenotypic traits, as is the case for *APOA1*: mutations in *APOA1* have been associated with an inability to activate LCAT and with hereditary amyloidosis [5]–[7]. While mutations that poorly activate LCAT associate with low apoA-I and HDL cholesterol levels due to the rapid removal of lipid-poor discoidal HDL from the circulation [30], mutations that cause amyloidosis may [5], [6] or may not [9]–[11] associate with low apoA-I and/or HDL cholesterol, most likely depending on the severity of the mutation [8]. Of the seventeen nonsynonymous variants identified in the present study, only seven have been reported by others (P4R, V11X, S36A, A37T, F71Y, K107del, L144R) [11], [13], [24], [27], [31]–[41]. Of these variants, five (P4R, V11X, S36A, K107del, L144R) associated with low apoA-I and/or HDL cholesterol levels in the CCHS and in other studies [13], [32]–[34], [40], [41]. L144R is unable to activate LCAT [13], while S36A, F71Y, K107del, have been associated with amyloidosis [24], [38]. A164S, a variant without any association with HDL cholesterol or apoA-I levels in the CCHS and CGPS, was associated with an increased risk of IHD, MI, and premature death, and with reduced survival after diagnosis of IHD in the CCHS, most likely due to an attenuated form of cardiac amyloidosis [13]. Thus, four variants in *APOA1* identified in 42 individuals (0.41% of the population) have either previously been associated with or suspected of causing amyloidosis. Only two of these variants, S36A and K107del, associated with low HDL cholesterol in the CCHS. This highlights two points: 1) The inherent weakness of the extreme approach when a gene has pleiotropic effects. Using this approach in the CCHS, both F71Y, a known amyloidosis mutation [24], and A164S, a suspected amyloidosis mutation associated with increased risk of ischemic heart disease and early mortality [13], would have been assumed to be nonfunctional; 2) That variants in *APOA1* associated with amyloidosis are relatively common in the general population.

Comparing the results from the population-based resequencing approach with the results obtained using only the extremes of the population distribution of apoA-I in the CCHS showed that NS variants were overrepresented in the lower percentiles of the apoA-I distribution, especially those predicted *in silico* to be more pathological, and correctly predicted association with low apoA-I and/or HDL cholesterol levels. Thus, 0.27% of the total population were heterozygous for one of nine different variants associated with substantial reductions in apoA-I and/or HDL cholesterol levels of up to, respectively, 39 mg/dL and 0.9 mmol/L.

As previously shown in the CCHS [13] and validated in the present study including the CGPS, NS mutations in *APOA1* may associate with increased risk of MI, without associating with reduced apoA-1 and HDL cholesterol levels. The most likley explanation for this is that these mutations represent less severe amyloidosis mutations manifesting clinically as MI, instead of the more severe restrictive cardiomyopathy [42]. Accordingly, we found that the combined NS mutations associated with an increased risk of MI, the main contributor to this increased risk was A164S, a presumed amyloidogenic mutation not associated with apoA-1 or HDL cholesterol levels in either the CCHS or the CGPS.

Our study suggests that the extreme phenotype approach used in a number of previous studies [12]–[16] is a powerful analytical strategy to capture the effects of both common and rare genetic variants on a specific *a priori* specified complex trait, provided the gene in question does not have pleiotropic effects. However, the success of this strategy depends on using a gene-near phenotype, preferably the direct gene product, on the size of the underlying study, the cut-off point for the extreme percentiles screened, and the frequencies and effect sizes of the identified variants.

In conclusion, population-based resequencing of *APOA1* identified a majority of rare NS variants associated with reduced apoA-I and HDL cholesterol levels and/or predisposing to amyloidosis. In addition, NS variants associated with increased risk of MI.

## Supporting Information

Figure S1 Plasma apoA-I and HDL cholesterol in percentiles for nonsynonymous and synonymous variants in *APOA1* stratified by gender. Data from the Copenhagen City Heart Study (CCHS) and the Copenhagen General Population Study (CGPS) combined. Each dot represents an individual with a given genetic variant. Percentiles corrected for age within each study, and stratified by gender. Solid black lines = median percentiles. Dashed line = 50^th^ percentile. P-values by Mann-Whitney U-test comparing females and males. Red, nonsynonymous variants identified exclusively in the lowest 20 percentile of the apoA-I distribution in the CCHS using the extreme phenotype approach; yellow, synonymous variants identified exclusively in the lowest 20 percentile; white boxes, nonsynonymous and synonymous variants identified in both the lowest and highest 20 percentiles.
(EPS)

Figure S2 Plasma levels of apoA-I and HDL cholesterol in percentiles for all synonymous variants in *APOA1*, and for nonsynonymous variants suspected of (A164S) or known to associate with amyloidosis (S36A-F71Y-L144R), or with reduced ability to activate LCAT (L144R). Data are from the Copenhagen City Heart Study (CCHS) and the Copenhagen General Population Study (CGPS) combined. Each dot represents an individual with a given genetic variant. Percentiles are corrected for gender and age within each study. Solid black lines = median percentiles. Dashed line = 50^th^ percentile. Percentiles between groups of variants are compared by Mann-Whitney U-test. P-values comparing median percentiles with 50^th^ percentile in the CCHS and CGPS as a whole by z-test and shown directly on figure. *P<0.05 and **P<0.01.
(EPS)

Table S1 PCR primers and fragment lengths for high-resolution melting analysis using a LightScanner followed by sequencing of *APOA1* in the Copenhagen City Heart Study.
(PDF)

Table S2 Genetic variation identified in the core promoter, coding region and exon-intron boundaries of *APOA1* in the Copenhagen City Heart Study (n = 10,330).
(PDF)

Table S3 Differences (Δ) in relative percentiles (50^th^ percentile in population minus heterozygote percentile) and in absolute levels (noncarriers minus heterozygotes) of apolipoprotein A-I and HDL cholesterol for *APOA1* variants in the Copenhagen City Heart Study and in the Copenhagen General Population Study.
(PDF)

Table S4 Lipid and lipoprotein levels in noncarriers and heterozygotes for synonymous(S) and nonsynonymous(NS) variants in the Copenhagen City Heart Study (left, top and bottom) and the Copenhagen General Population Study (right, bottom).
(PDF)
