# Supplementary material for: Population-Based Resequencing of APOA1 in 10,330 Individuals: Spectrum of Genetic Variation, Phenotype, and Comparison with Extreme Phenotype Approach
Source: PLoS Genet. 2012 Nov 29;8(11):e1003063. doi: 10.1371/journal.pgen.1003063 (PMC3510059; doi:10.1371/journal.pgen.1003063)
Supplement: Table S1 — PCR primers and fragment lengths for high-resolution melting analysis using a LightScanner followed by sequencing of APOA1 in the Copenhagen City Heart Study. (PDF) [file pgen.1003063.s003.pdf]

Table S1: PCR primers and fragment lengths for high-resolution melting analysis using a LightScanner followed by sequencing of *APOA1* in the Copenhagen City Heart Study.

APOA1	Forward primer<br>5’ → 3’	Reverse primer<br>5’ → 3’	Fragment length
Exon 2	IVS1-119’TACCTGAGGTCTTCTCCCGCTCT	IVS2+35’CCGATGGTTGGCTCCCTAGGTTA	217 bp
Exon 3	IVS2-38’ACAGCTGGCCTGATCTG	IVS3+17’CCAGGCTGGGTCCTTAC	212 bp
Exon 4a	IVS3-30’AGCCCTCAACCCTTCTGTCTCAC	c.506’CCCAGTGGGCTCAGCTTCTCTTG	336 bp
Exon 4b	c.432’GCCGCTGCGCGCAGAGCTCCAAGAGGG	*44’AAACGTTTATTCTGAGCACCGGGAAGG	418 bp

The primers were designed using Oligo 6.71 (Molecular Biology Insights Inc., CO, USA) and the *APOA1* consensus sequence NC_000011.9. The start site for each primer is indicated.
