# Supplementary material for: Population-Based Resequencing of APOA1 in 10,330 Individuals: Spectrum of Genetic Variation, Phenotype, and Comparison with Extreme Phenotype Approach
Source: PLoS Genet. 2012 Nov 29;8(11):e1003063. doi: 10.1371/journal.pgen.1003063 (PMC3510059; doi:10.1371/journal.pgen.1003063)
Supplement: Table S2 — Genetic variation identified in the core promoter, coding region and exon-intron boundaries of APOA1 in the Copenhagen City Heart Study (n = 10,330). (PDF) [file pgen.1003063.s004.pdf]

Table S2. Genetic variation identified in the core promoter, coding region and exon-intron boundaries of *APOA1* in the Copenhagen City Heart Study (n=10,330).

Gene Region	Nucleotide Substitution[a]	Amino Acid Residue	No. of individuals in CCHS, n=10,330 (minor allele frequency,%)	Functional Region/Function	Previous reports/ rs numbers
Promoter	g.-647A>G	-	106 (0.5)	192 bp 5' of regulatory region D (HRE)[b]	-
Promoter	g.-560A>C	-	718 (3.5)	105 bp 5' of regulatory region D (HRE)[b]	[1]/rs12718466
Promoter	g.-310G>A	-	3,043 (16.0)	2 bp 3' of regulatory region B (HRE)[b]	[1]/rs670
Intron 1	g.-151C>T	-	712 (3.5)	67 bp 3' of regulatory region A[b]	[1]/rs5069
Intron 1	g.-150G>A	-	143 (0.7)	68 bp 3' of regulatory region A[b]	rs1799837
Intron 1	g.-108_-106delCTC	-	3 (0.01)	111 bp 3' of regulatory region A[b]	rs61758321
Intron 1	g.-89G>A	-	7 (0.03)	130 bp 3' of regulatory region A[b]	-
Intron 1	g.-35_-33delCTT	-	1 (0.005)	184 bp 3' of regulatory region A[b]	-
Intron 1	g.-11G>A	-	12 (0.06)	208 bp 3' of regulatory region A[b]	-
Exon2	c. 9T>C	A(-22)A	1 (0.005)	Prepeptide[c]	-
Exon 2	c. 14T>C	V(-20)A	1 (0.005)	Prepeptide[c]	-
Intron 2	IVS2+41C>T		(30.0)[d]	-	[1]/rs5070
Exon 3	c. 83C>G	P4R	2 (0.01)	Amino-terminal end	[2,3]

Exon 3	c. 79delC	V11X	1 (0.005)	Helix 1	[4,5]
Exon 3	c. 108G>A	K12K	4 (0.02)	Helix 1	-
Exon 3	c. 147C>T	S25S	3 (0.01)	Between helix 1 and 2 – potential ESE	-
Exon 3	c. 176G>T	G35V	1 (0.005)	Between helix 1 and 2	-
Exon 3	c. 178T>G	S36A	5 (0.02)	Between helix 1 and 2	[6,7]
Exon 3	c. 181G>A	A37T	2 (0.01)	Between helix 1 and 2	[1,8,9] /rs12718465
Intron 3	IVS3+33C>T		(6.0)[d]	-	[1]/rs2070665
Exon 4	c.284T>A	F71Y	9 (0.04)	Induction of hypertriglyceridemia[e] and amyloidosis[f]	[7]
Exon 4	c. 294C>A	N74K	1 (0.005)	Induction of hypertriglyceridemia[e] and amyloidosis[f]	-
Exon 4	c. 298G>C	E76Q	2 (0.01)	Induction of hypertriglyceridemia[e] and amyloidosis[f]	-
Exon 4	c. 391delAAG	K107del	4 (0.02)	Induction of hypertriglyceridemia[e]	[10-14]
Exon 4	c.498C>A	S142R	1 (0.005)	Helix 5	-
Exon 4	c.503T>G	L144R	4 (0.02)	Activation of LCAT[e]	[4,15]
Exon 4	c.524G>A	R151H	1 (0.005)	Activation of LCAT[e]	-
Exon 4	c.526G>A	A152T	1 (0.005)	Activation of LCAT[e]	-
Exon 4	c.529C>T	R153C	1 (0.005)	Activation of LCAT[e]	-
Exon 4	c.562G>T	A164S	24 (0.1)	Activation of LCAT[e]	[4]
Exon 4	c.564G>G	A164A	2 (0.01)	Activation of LCAT[e] – potential ESE	-

Exon 4	[c.572G>A;c.753C>A]	S167L	2 (0.01)	Activation of LCAT[e]	-
Exon 4	c.642C>G	A190A	5 (0.02)	Helix 8 – potential ESE	-
Exon 4	c.669T>C	H199H	1 (0.005)	Helix 8 – potential ESE	-
Exon 4	c.732C>G	P220P	2 (0.01)	Interaction with ABCA1[e] – potential ESE	rs5080
3’UTR	*8G>A		4 (0.02)	Potential ESE	-
3’UTR	*17C>T		7 (0.03)	-	-
3’UTR	*141G>A		33 (0.2)	-	-
Intergenic	*178T>A		(1.6)[d]	-	[1]/rs5081
Intergenic	*181A>G		(3.5)[d]	-	[1]/rs12718463

In shaded grey, genetic variants identified by screening the regulatory and coding regions of *APOA1* in 95 individuals with extreme low apoA-I levels and 95 individuals with extreme high apoA-I levels in the Copenhagen City Heart Study followed by genotyping in the whole population [16]. [a]Nucleotide +1 denotes A in the start codon ATG (*translational* start site) in exon 2 (NM_000039.1), corresponding to base position 236 in *APOA1* consensus sequence NC_000011.9; to convert to nucleotide position relative to the *transcriptional* start site add 235 nucleotides. [b]Four regulatory regions, A-D, have been reported in the *APOA1* promoter, of which regions B and D are hormone responsive elements (HRE) that among others bind hepatic nuclear factor 4; element C binds CCAAT enhancer binding protein [17]. [c]Full-length apoA-I (267 amino acids) includes a preprotein (18 amino acids) and a proprotein (6 amino acids), which are consecutively cleaved to form the mature protein (243 amino acids). [d]Frequency of genetic variants determined based on screening the regulatory and coding regions of *APOA1* in 95 individuals with extreme low apoA-I levels and 95 individuals with extreme high apoA-I levels in the Copenhagen City Heart Study [16]. [e]Reference [18]. [f]Reference [19]. ABCA1 = ATP Binding Casette Transporter A1; ESE = exonic splicing enhancer region predicted by use of “Esefinder” (http://rulai.cshl.edu/cgi-bin/tools/ESE3/esefinder.cgi). LCAT = Lecithin:cholesterol acyltransferase.

REFERENCES S1

1. Fullerton M, Buchanan V, Sonpar A, Taylor L, Smith D et al. (2004) The effects of scale: variation in the APOA1/C3/A4/A5 gene cluster. Hum Genet V115: 36-56.

2. Menzel HJ, Assmann G, Rall SC, Jr., Weisgraber KH, Mahley RW (1984) Human apolipoprotein A-I polymorphism. Identification of amino acid substitutions in three electrophoretic variants of the Münster-3 type. J Biol Chem 259: 3070-3076.

3. von Eckardstein A, Funke H, Henke A, Altland K, Benninghoven A et al. (1989) Apolipoprotein A-I variants. Naturally occurring substitutions of proline residues affect plasma concentration of apolipoprotein A-I. J Clin Invest 84: 1722-1730.

4. Haase CL, Frikke-Schmidt R, Nordestgaard BG, Kateifides AK, Kardassis D et al. (2011) Mutation in APOA1 predicts increased risk of ischaemic heart disease and total mortality without low HDL cholesterol levels. J Int Med 270: 136-146.

5. Miccoli R, Bertolotto A, Navalesi R, Odoguardi L, Boni A et al. (1996) Compound heterozygosity for a structural apolipoprotein A-I variant, apo A-I(L141R)Pisa, and an apolipoprotein A-I null allele in patients with absence of HDL cholesterol, corneal opacifications, and coronary heart disease. Circulation 94: 1622-1628.

6. Kiss RS, Kavaslar N, Okuhira Ki, Freeman MW, Walter S et al. (2007) Genetic etiology of isolated low HDL syndrome: Incidence and heterogeneity of efflux defects. Arterioscler Thromb Vasc Biol 27: 1139-1145.

7. Rowczenio D, Dogan A, Theis JD, Vrana JA, Lachmann HJ et al. (2011) Amyloidogenicity and clinical phenotype associated with five novel mutations in apolipoprotein A-I. Am J Pathol 179: 1978-1987.

8. Araki K, Sasaki J, Matsunaga A, Takada Y, Moriyama K et al. (1994) Characterization of two new human apolipoprotein A-I variants: apolipoprotein A-I Tsushima (Trp-108->Arg) and A-I Hita (Ala-95->Asp). Biochim Biophys Acta 1214: 272-278.

9. Matsunaga T, Hiasa Y, Yanagi H, Maeda T, Hattori N et al. (1991) Apolipoprotein A-I deficiency due to a codon 84 nonsense mutation of the apolipoprotein A-I gene. Proc Natl Acad Sci USA 88: 2793-2797.

10. Rall SC Jr., Weisgraber KH, Mahley RW, Ogawa Y, Fielding CJ et al. (1984) Abnormal lecithin:cholesterol acyltransferase activation by a human apolipoprotein A-I variant in which a single lysine residue is deleted. J Biol Chem 259: 10063-10070.

11. Utermann G, Feussner G, Franceschini G, Haas J, Steinmetz A (1982) Genetic variants of group A apolipoproteins. Rapid methods for screening and characterization without ultracentrifugation. J Biol Chem 257: 501-507.

12. Amarzguioui M, Mucchiano G, Häggqvist B, Westermark P, Kavlie A et al. (1998) Extensive intimal apolipoprotein A1-derived amyloid deposits in a patient with an apolipoprotein A1 mutation. Biochem Biophys Res Commun 242: 534-539.

13. Tilly-Kiesi M, Packard CJ, Kahri J, Ehnholm C, Shepherd J et al. (1997) In vivo metabolism of apoA-I and apoA-II in subjects with apo A-I(Lys107->0) associated with reduced HDL cholesterol and Lp(AI w AII) deficiency. Atherosclerosis 128: 213-222.

14. Tilly-Kiesi M, Qiuping Z, Ehnholm S, Kahri J, Lahdenpera S et al. (1995) ApoA-IHelsinki (Lys107->0) associated with reduced HDL cholesterol and LpA-I:A-II deficiency. Arterioscler Thromb Vasc Biol 15: 1294-1306.

15. Recalde D, Velez-Carrasco W, Civeira F, Cenarro A, Gomez-Coronado D et al. (2001) Enhanced fractional catabolic rate of apo A-I and apo A-II in heterozygous subjects for apo A-IZaragoza (L144R). Atherosclerosis 154: 613-623.

16. Haase CL, Tybjærg-Hansen A, Grande P, Frikke-Schmidt R (2010) Genetically elevated apolipoprotein A-I, high-density lipoprotein cholesterol levels, and risk of ischemic heart disease. J Clin Endocrinol Metab 95: E500-E510.

17. Zannis VI, Kan HY, Kritis A, Zanni EE, Kardassis D (2001) Transcriptional regulatory mechanisms of the human apolipoprotein genes in vitro and in vivo. Curr Opin Lipidol 12: 181-207.

18. Zannis VI, Koukos G, Drosatos K, Vezeridis A, Zanni EE et al. (2008) Discrete roles of apoA-I and apoE in the biogenesis of HDL species: Lessons learned from gene transfer studies in different mouse models. Ann Med 40 Suppl 1: 14-28.

19. Eriksson M, Schönland S, Yumlu S, Hegenbart U, von Hutten H et al. (2009) Hereditary apolipoprotein AI-associated amyloidosis in surgical pathology specimens. Identification of three novel mutations in the APOA1 gene. J Mol Diagn 11:257-262.
