# Supplementary material for: Population-Based Resequencing of APOA1 in 10,330 Individuals: Spectrum of Genetic Variation, Phenotype, and Comparison with Extreme Phenotype Approach
Source: PLoS Genet. 2012 Nov 29;8(11):e1003063. doi: 10.1371/journal.pgen.1003063 (PMC3510059; doi:10.1371/journal.pgen.1003063)
Supplement: Table S3 — Differences (Δ) in relative percentiles (50th percentile in population minus heterozygote percentile) and in absolute levels (noncarriers minus heterozygotes) of apolipoprotein A-I and HDL cholesterol for APOA1 variants in the Copenhagen City Heart Study and in the Copenhagen General Population Study. (PDF) [file pgen.1003063.s005.pdf]

Table S3. Differences (Δ) in relative percentiles (50th percentile in population minus heterozygote percentile) and in absolute levels (noncarriers minus heterozygotes) of apolipoprotein A-I and HDL cholesterol for *APOA1* variants in the Copenhagen City Heart Study and in the Copenhagen General Population Study.

APOA1 variant	Median difference in apolipoprotein A-I				Median difference in HDL cholesterol			
	ΔPercentiles (50th percentile–heterozygotes)	P-value	ΔAbsolute levels (mg/dL) (noncarriers – heterozygotes)	P-value	ΔPercentiles (50th percentile in – heterozygotes)	P-value	ΔAbsolute levels (mg/dL) (noncarriers – heterozygotes)	P-value
K12K								
CCHS n=4	11 (-23 to 32)		8 (-36 to 38)		19 (2 to 36)		0.22 (0 to 0.52)	
CGPS n=16	18 (-1 to 35)	0.67	16 (8 to 24)	0.29	13 (-11 to 30)	0.60	0.18 (0 to 0.37)	0.77
S25S								
CCHS n=3	6 (-24 to 46)		14 (-30 to 53)		4 (-50 to 48)		0.10 (-2.00 to 0.90)	
CGPS n=24	16 (-26 to 38)	0.94	8 (-6 to 21)	0.74	8 (-30 to 37)	0.84	0.05 (-0.20 to 0.28)	0.31
S36A								
CCHS n=5	40 (36 to 42)		29 (18 to 40)		36 (24 to 43)		0.40 (0.30 to 0.50)	
CGPS n=29	26 (12 to 43)	0.51	11 (4 to 19)	0.19	21 (-7 to 31)	0.07	0.06 (-0.08 to 0.20)	0.11
F71Y								
CCHS n=9	-23 (-29 to 11)		-16 (-31 to 1)		-8 (-15 to 8)		-0.20 (-0.30 to 0.00)	
CGPS n=48	19 (-6 to 39)	0.02	13 (6 to 19)	0.01	21 (0 to 35)	0.04	0.20 (0.07 to 0.32)	0.09
K107del								
CCHS n=4	27 (1 to 35)		16 (-8 to 39)		45 (26 to 46)		0.40 (0.10 to 0.70)	
CGPS n=9	-22 (-35 to 5)	0.03	-10 (-21 to 2)	0.17	33 (15 to 38)	0.31	0.40 (0.14 to 0.66)	0.82
L144R								
CCHS n=4	47 (39 to 49)		41 (22 to 60)		48 (49 to 46)		0.70 (0.60 to 0.80)	
CGPS n=12	45 (43 to 49)	0.80	47 (34 to 61)	0.64	48 (49 to 46)	0.90	0.85 (0.70 to 1.01)	0.65
A164S								
CCHS n=24	-7 (-30 to 21)		-2 (-14 to 8)		4 (-32 to 26)		0.00 (-0.20 to 0.20)	
CGPS n=108	-1 (-28 to 22)	0.59	-1 (-6 to 4)	0.50	-3 (-29 to 17)	0.59	-0.04 (-0.12 to 0.05)	0.70
A190A								
CCHS n=5	22 (4 to 39)		17 (-1 to 36)		0 (-3 to 39)		0.20 (-0.10-0.50)	
CGPS n=16	-7 (-26 to 16)	0.09	-2 (-14 to 11)	0.22	-2 (-22 to 17)	0.32	0.04 (-0.13-0.22)	0.50

Differences in relative percentiles are shown as medians (interquartile range) between the 50th percentile in the population and heterozygotes for a given variant, and differences in absolute levels as median differences (95% confidence interval) between noncarriers and heterozygotes in the Copenhagen City Heart Study (CCHS) and the Copenhagen General Population Study (CGPS). P-values comparing relative percentile differences and absolute differences in the CCHS and CGPS by, respectively, Mann-Whitney U-test and z-test.
