# Supplementary material for: Population-Based Resequencing of APOA1 in 10,330 Individuals: Spectrum of Genetic Variation, Phenotype, and Comparison with Extreme Phenotype Approach
Source: PLoS Genet. 2012 Nov 29;8(11):e1003063. doi: 10.1371/journal.pgen.1003063 (PMC3510059; doi:10.1371/journal.pgen.1003063)
Supplement: Table S4 — Lipid and lipoprotein levels in noncarriers and heterozygotes for synonymous(S) and nonsynonymous(NS) variants in the Copenhagen City Heart Study (left, top and bottom) and the Copenhagen General Population Study (right, bottom). (PDF) [file pgen.1003063.s006.pdf]

Table S4. Lipid and lipoprotein levels in noncarriers and heterozygotes for synonymous(S) and nonsynonymous(NS) variants in the Copenhagen City Heart Study (left, top and bottom) and the Copenhagen General Population Study (right, bottom).

	CCHS				CGPS			
	Noncarriers	Heterozygotes for S	Heterozygotes for all NS	Heterozygotes for NS without A164S	Noncarriers	Heterozygotes for S	Heterozygotes for all NS	Heterozygotes for NS without A164S
**Variants typed in CCHS: A(-22)A, V(-20)A, P4R, V11X, K12K, S25S, G35V, S36A, A37T, F71Y, N74K, E76Q, K107del, S142R, L144R, R151H, A152T, R153C, A164S, A164A, S167L, A190A, H199H, P220P**								
No. of individuals	10,251	18	61	38				
Age (years)	59 (45-69)	65 (47-68)	59 (44-68)	61 (43-71)				
Sex (M/F)	4,540/5,711	9/9	28/33	15/23				
Apolipoprotein A-I (mg/dL)	140 (123-161)	129 (103-149)	137 (115-161)	130 (107-162)				
HDL cholesterol (mmol/L)	1.5 (1.2-1.8)	1.3 (0.9-1.7)	1.4 (1.1-1.7)[a]	1.3 (1.0-1.7)[a]				
Total cholesterol (mmol/L)	6.0 (5.1-6.9)	5.8 (5.3-6.1)	6.0 (5.4-6.7)	6.0 (5.7-6.7)				
LDL cholesterol (mmol/L)	3.6 (2.9-4.4)	3.5 (3.0-3.7)	3.7 (3.1-4.3)	3.8 (3.3-4.4)				
Triglycerides (mmol/L)	1.5 (1.1-2.2)	1.6 (1.2-2.4)	1.8 (1.3-2.4)	1.8 (1.5-2.4)[a]				
Lipid lowering therapy (%)	1	0	2	0				
**Variants typed in CCHS and CGPS: K12K, S25S, S36A, F71Y, K107del, L144R, A164S, A190A**								
No. of individuals	10,272	12	46	22	44,977	56	206	98
Age (years)	59 (45-69)	65 (49-67)	60 (44-67)	63 (43-68)	57 (47-67)	58 (47-68)	57 (48-66)	56 (46-68)
Sex (M/F)	4,549/5,723	7/5	21/25	7/15	20,210/24,767	29/27	86/120	31/67[b]
Apolipoprotein A-I (mg/dL)	140 (123-161)	129 (103-143)	137 (117-154)	126 (106-162)	156 (139-176)	145 (130-173)[a]	151 (134-168)[b]	141 (129-162)[b]
HDL cholesterol (mmol/L)	1.5 (1.2-1.8)	1.3 (1.0-1.6)	1.4 (1.1-1.7)	1.2 (1.0-1.6)[b]	1.6 (1.3-2.0)	1.5 (1.2-1.8)	1.5 (1.2-1.8)[b]	1.3 (1.0-1.6)[b]
Total cholesterol (mmol/L)	6.0 (5.1-6.9)	5.8 (5.4-6.0)	6.2 (5.0-6.9)	6.2 (5.0-6.9)	5.6 (4.9-6.3)	5.4 (4.7-6.2)	5.5 (4.8-6.1)	5.5 (4.7-6.1)
LDL cholesterol (mmol/L)	3.6 (2.9-4.4)	3.5 (3.0-3.7)	3.7 (2.9-4.5)	3.7 (3.1-4.5)	3.2 (2.6-3.8)	3.0 (2.6-3.9)	3.2 (2.6-3.8)	3.3 (2.7-3.8)
Triglycerides (mmol/L)	1.5 (1.1-2.2)	1.5 (1.2-2.2)	1.8 (1.2-2.2)	1.8 (1.5-2.4)	1.4 (1.0-2.1)	1.5 (1.0-2.3)	1.4 (0.9-2.1)	1.5 (0.9-2.4)
Lipid lowering therapy (%)	1	0	1	2	10	9	12	12

Values are median (interquartile range) or percentage for noncarriers and heterozygotes for S and NS variants. Kruskal-Wallis test with Mann-Whitney U as a post hoc test or Fishers exact test was used for continuous and categorical traits, respectively. LDL cholesterol levels were calculated using the Friedewald equation if triglycerides were $\leq$4 mmol/L, but measured directly at higher triglyceride levels. Lipid-lowering therapy was self-reported. [a]$P<0.05$ and [b]$P<0.01$ for heterozygotes versus noncarriers.
